# Effects of artificial intelligence based physiotherapy educational approach in developing clinical reasoning skills: a randomized controlled trial

**DOI:** 10.1186/s12909-025-07926-w

**Published:** 2025-10-09

**Authors:** Gizem Ergezen Sahin, Gulay Aras Bayram, Alberto Sanchez Sierra, Simay Akdemir, Dogukan Kurc, Devrim Tarakci, Ayse Nur Tunali

**Affiliations:** 1 Faculty of Health Sciences, Physiotherapy and Rehabilitation, Istanbul Medipol University, Beykoz, Istanbul, 34810 Türkiye; 2 Grupo de Investigación en Fisioterapia Toledo (GIFTO), Facultad de Fisioterapia y Enfermería, Universidad de Castilla-La Mancha, Toledo, Spain; 3 Grupo de Investigación en Fisioterapia Toledo (GIFTO), Instituto de Investigación Sanitaria de Castilla-La Mancha, Toledo, Spain; 4 Faculty of Health Sciences, Ergotherapy, Istanbul Medipol University , Istanbul, Türkiye

**Keywords:** Artificial intelligence, Problem-based learning, Physiotherapy education, Clinical competence, AI self-efficacy, Reading motivation, Internet addiction

## Abstract

**Background:**

Artificial intelligence (AI) tools such as ChatGPT are increasingly being integrated into health professions education, but evidence regarding their application in physiotherapy remains limited. This study aims to investigate the impact of AI-assisted problem-based learning (AI-PBL) on theoretical knowledge, clinical competence, AI self-efficacy, internet addiction, and reading motivation compared with traditional PBL.

**Methods:**

A randomized controlled trial was conducted with undergraduate physiotherapy students assigned to AI-PBL or PBL groups. Participants completed assessments before, immediately after, and two weeks after the group intervention. Outcome measures included a theoretical knowledge test, the Mini Clinical Evaluation Exercise (Mini-CEX), the AI ​​Self-Efficacy Scale (AI-SES), the Internet Addiction Test (IAT), and the Adult Reading Motivation Scale (ARMS).

**Results:**

Forty students were randomized equally into two groups: AI-PBL (*n* = 20) and traditional PBL (*n* = 20). Both groups showed significant improvements in knowledge and reading motivation. The AI-PBL group showed significantly greater improvement in knowledge retention at 2 weeks (Cohen’s *d* = 3.14) and greater gains in AI self-efficacy. Although Mini-CEX scores were higher in the AI-PBL group, the differences between groups were not statistically significant. No significant increase in internet addiction was observed in the AI-PBL group.

**Conclusion:**

These findings emphasize that supervised, structured use of generative AI in education can enhance sustained learning and digital self-efficacy without posing behavioral risks. The AI-PBL approach appears to foster active reflection, self-directed learning, and deeper academic engagement offering a promising direction for digital innovation in physiotherapy education. Future studies should explore long-term outcomes, track behavioral engagement, and further validate the benefits of AI-enhanced instructional strategies.

**Trial registration:**

Prior to the initiation of the study, the protocol was registered on https//www.clinicaltrials.gov/, and registration status was made publicly available (Identifier NCT07010991 Date 08.06.2025).

(https://clinicaltrials.gov/study/NCT07010991?term=NCT07010991&rank=1).

**Supplementary Information:**

The online version contains supplementary material available at 10.1186/s12909-025-07926-w.

## Background

With the rapid integration of artificial intelligence (AI) into daily life, individuals have begun to benefit from this technology as a part of their routines in various domains such as healthcare, education, and transportation. AI is considered as one of the most transformative technologies of the 21 st century due to its potential to enhance efficiency and improve quality of life across society [1].

In healthcare, AI offers significant advantages including improving diagnostic accuracy, enhancing efficiency in clinical workflows, generating personalized treatment plans, supporting data analysis in technology-assisted rehabilitation, and reducing the administrative burden on healthcare professionals. However, concerns have also been raised regarding the ethical and potential risks of this technology, such as biased algorithms, lack of transparency in decision-making processes, and the security of patient data [2, 3].

Similarly, in the field of health professions education, AI has gained prominence particularly through applications such as monitoring student performance via learning analytics tools, creating personalized learning pathways, and leveraging large datasets [4]. While these technologies offer significant opportunities for designing learner-centered educational experiences, Feigerlova et al. emphasize that educators must critically evaluate AI-generated content in terms of learning outcomes and support students’ independent thinking and clinical reasoning skills within the curriculum. Moreover, it has been noted that scientific evidence regarding the effectiveness of AI-supported educational applications on learning outcomes is still insufficient [5]. Physiotherapy and rehabilitation education aims not only to convey theoretical knowledge but also to develop multifaceted skills such as clinical decision-making, problem-solving, and effective communication with patients. For this purpose, the problem-based learning (PBL) model, which is an evidence-based and student-centered approach, is frequently adopted [6]. Importantly, it also requires the development of hands-on practical competencies, which constitute both a unique challenge and a key advantage in physiotherapy training, as they are essential for preparing students to meet real-world clinical demands [7]. However, traditional PBL practices can be time-consuming and instructor-dependent, particularly in terms of developing case scenarios and accessing information. At this point, integrating AI-supported tools such as ChatGPT into PBL processes may offer positive contributions in terms of student performance, learning satisfaction, engagement, and efficient use of time [8, 9]. Nonetheless, Lindbäck et al. (2025) suggest that while these tools can enrich physiotherapy education when appropriately guided and supported by critical thinking scaffolds, uncontrolled use may pose risks for learning outcomes [10]. Similarly, Sallam et al. highlight that the effective use of AI in health education requires proactive risk management, stressing the importance of cautious application of widely used AI tools such as ChatGPT despite their transformative potential. In this context, AI literacy training is considered a key strategy for managing such risks effectively [11].

Recent literature suggests that ChatGPT-based approaches can enhance theoretical knowledge acquisition and contribute to the development of clinical competencies in medical education [12, 13]. However, such applications remain underexplored in the specific context of physiotherapy and rehabilitation. Particularly in conditions like chronic low back pain (CLBP) which demands a biopsychosocial approach, multidimensional assessment, and complex clinical reasoning, ChatGPT-supported learning through personalized case scenarios is proposed to offer substantial educational benefits [14]. ChatGPT has been observed to actively engage students in processes of understanding, analyzing, and applying information through structured and guiding prompts. These AI-assisted learning modules not only facilitate rapid access to information but also support the development of critical thinking and interpretive skills, thereby deepening the overall learning experience. Nevertheless, to ensure that such educational processes are conducted effectively and ethically, it is crucial for students to be equipped with AI literacy and for educators to guide these tools’ integration into curricular content [14]. This approach may foster more informed and effective use of AI technologies in health professions education.

The aim of this study was to evaluate the impact of an AI-supported problem-based learning module on physiotherapy undergraduate students’ theoretical knowledge, clinical decision-making skills, and perceived self-efficacy in the use of AI, and to compare these outcomes with those of a traditional PBL approach.

## Materials and methods

### Study design and settings

This study was a prospective, two-arm, parallel-group, randomized controlled trial conducted in June 2025. The trial aimed to evaluate the effectiveness of an AI-supported PBL module, powered by ChatGPT, compared to a traditional instructor-led PBL approach among undergraduate physiotherapy students. The trial was conducted simultaneously at two institutions: Istanbul Medipol University, Turkiye and Universidad de Castilla-La Mancha, Spain. This study, which was carried out in line with the Declaration of Helsinki’s ethical framework. Ethical approval for the study was obtained from the Institutional Review Board of Istanbul Medipol University on May 8, 2025 (Approval No: E-10840098-202.3.02-2975/536). All participants received comprehensive information regarding the study’s purpose, methodology, potential risks, and benefits, and subsequently provided written informed consent prior to enrollment. All collected data were anonymized, securely stored, and used exclusively for research purposes. The study did not include any vulnerable populations or invasive procedures. The study adhered to the CONSORT 2025 guidelines for randomized trials in educational research.

### Participants

A total of 94 s-year undergraduate physiotherapy students who had an internship experience before, were recruited on a voluntary basis. An a priori power analysis was conducted using G*Power 3.1, with reference to outcome measures from Hui et al. (2025), which reported significant post-intervention improvements in theoretical knowledge scores following ChatGPT-assisted PBL [8]. To detect a moderate effect size with 80% power and a significance threshold of 0.05, a sample size of at least 20 participants per group was deemed adequate. Participants were randomly allocated into two groups after initial theoretical and practical sessions: the AI-supported PBL group (AI-PBLG) and the traditional instructor-led PBL group (PBLG). To ensure randomization and allocation concealment, participants were assigned to the AI-PBLG or traditional PBLG group using sealed opaque envelopes. An independent researcher prepared sequentially numbered, identical envelopes containing group allocations based on a computer-generated randomization list. Allocation concealment was ensured until the moment of assignment.

### Blinding

Due to the nature of the educational intervention, blinding of participants and instructors was not feasible. However, outcome assessors responsible for the Mini clinical evaluation exercise (Mini-CEX) and the data analyst performing the statistical analyses were blinded to group allocation to minimize potential bias.

### Eligibility criteria

Eligible participants were second-year undergraduate students with no prior exposure to AI-supported PBL activities or PBL-based training who had formally enrolled in the CLBP module, successfully completed a preliminary theoretical knowledge assessment, and voluntarily provided written informed consent to participate.

Participants were excluded if they previously attended a course that included AI-supported PBL instruction and did not accept to give informed consent at the beginning of the study. Also the freshmans who do not have any knowledge about the practice in physiotherapy and the seniors who have a lot of practical experience at the hospital internship were excluded from the study. During the study ongoing process, study exclusion criteria were, attending to less than 80% of the total 8-h educational intervention and failing to complete pre- or post-intervention assessments. Students who refused to allow their anonymized data to be used for research purposes or encountered technical barriers such as lack of access to a digital device or the internet during the AI-based learning sessions were also excluded from the study. Although students who were excluded from the study continued their education, their data were not included in the analysis. Students participated on a voluntary basis and were clearly informed that they could withdraw from the study at any time without any impact on their academic standing.

### Intervention

An open announcement was made to all students at Istanbul Medipol University and Universidad de Castilla-La Mancha, after which volunteers completed an online application form that also collected demographic and academic information. Following this, participants undertook a baseline theoretical knowledge test (T0, pre-test) consisting of 20 multiple-choice questions on CLBP, covering anatomy, pathophysiology, clinical presentation, assessment, and management. Each correct response was worth five points (maximum score 100), with scores below 50 considered unsuccessful. Students who achieved ≥ 50 and met all inclusion criteria were enrolled. The same exam was subsequently repeated immediately after the initial workshop (T1, immediate post-test) and at the end of the two-week intervention (T2, final post-test). Secondary outcomes (Internet Addiction Test, the Adult Reading Motivation Scale, and the Artificial Intelligence Self-Efficacy Scale) were administered at T0 and T2, while the Mini-CEX was conducted only once, at T2.

The educational intervention consisted of an 8-hour instructional module on CLBP rehabilitation, delivered over two weeks (4 h per week). In the first week, students attended a 2-hour theoretical lecture and a 45-minute practical demonstration, followed by random allocation into either the AI-PBLG or PBLG groups. The remaining 75 min of the session were dedicated to group-specific instruction. In the second week, students participated in a 4-hour session focused on active application, including subgroup presentations of clinical case analyses, simulation-based discussions, and submission of a written group report.

The learning objectives for both groups were structured according to the cognitive domain of Bloom’s taxonomy, which provides a hierarchical classification of learning processes. This framework organizes cognitive skills into six progressive levels: remembering, understanding, applying, analyzing, evaluating, and creating, thereby facilitating the design of clear and measurable educational outcomes [15] (Table 1).

**Table 1:** Course objectives for AI-PBLG and PBLG

Bloom Level	AI-PBLG	PBLG
Information/ Remembering	Describe the definition, classifications, and risk factors for CLBP.	Describe the definition, classifications, and risk factors for CLBP.
Understanding	Explains the approaches to history taking, assessment and exercise in CLBP; interprets the biopsychosocial model.	Explains the approaches to history taking, assessment and exercise in CLBP; interprets the biopsychosocial model.
Applying	Creates a structured virtual case scenario that includes patient history, symptoms, assessment, and treatment using AI.	Applies evaluation and exercises in case examples under the guidance of the instructor.
Analyzing	Compares different treatment options; analyzes the accuracy of information received from AI.	Classifies physical findings and takes them into consideration in exercise selection.
Creating	Creates a unique case scenario and individualized treatment plan with AI.	Creates a sample exercise plan individually or in groups based on the case content presented under the guidance of the instructor.
Evaluating	Critically evaluates own learning process and contributions of using AI. Compares information obtained from AI with scientific sources and analyzes content accuracy. Questions about knowledge gaps or contradictions that arise during interaction with AI.	Compares different assessment and treatment options presented in case discussions and justifies which is most clinically appropriate.

*AI-PBLG* Artificial intelligence–supported problem-based learning group, *PBLG* Traditional problem-based learning group, *CLBP* Chronic low back pain

### Theoretical knowledge assessment

All participants completed a 20-item multiple-choice theoretical knowledge exam at three time points: (T0) prior to the initial theoric session (pre-test), (T1) immediately following the initial theoretical session (immediate post-test), and (T2) after completion of the 2-week intervention (post-test). Each test was scored on a 0–100 scale, with a score below 50 considered as unsuccessful. The exam content was developed in alignment with the educational objectives and reviewed by content experts in musculoskeletal physiotherapy. The test items were designed to assess factual knowledge, conceptual understanding, and clinical reasoning related to CLBP. The same exam was used across both groups to ensure consistency and comparability.

### Initial theoretical session

Following random assignment, both groups received a standard introductory course supported by multimedia displays consisting of text, audio, video, and visuals covering the core concepts of CLBP. The following topics were included:

1. Overview of Lumbar disc herniation-related low back pain.
2. Anatomy of Low back pain associated with lumbar disc herniation.
3. Pathophysiology of disc herniation.
4. Classification systems for lumbar disc herniation.
5. Clinical presentation and diagnostic approaches.
6. Evidence-based treatment and management strategies.

At the end of the theoric section, all students received digital access to supplementary learning materials, including two narrative reviews, two clinical guidelines, two textbook chapters, and the slide presentations used during the session. In order to further evaluate the contribution of theoretical education, the same theoretical exam was administered to the students for the second time.

### Practical demonstration session

Following the initial 2-hour theoretical instruction, all participants attended a 45-minute live demonstration of manual therapy techniques widely utilized in the clinical management of chronic low back pain. These techniques included Mulligan mobilizations, kinesiotaping applications, soft tissue mobilization, stretching routines, and classical massage techniques. The demonstrations were performed by the instructor on an assistant and were standardized for both groups. To ensure consistency, avoid intervention overlap between groups, and observe students’ manual skill development by themselves, students were not allowed to engage in hands-on practice during this session.

### Initial case assignment

Following the theoretical and practical introductory sessions, participants were randomized into two instructional arms: the AI–supported PBL group (AI-PBLG) and the traditional PBL group (PBLG). The two groups received instruction simultaneously in separate classrooms. Both groups were presented with the same initial clinical case scenario (Appendix 1), and were asked to collaboratively develop and report a treatment plan based on the theoretical content covered in the initial sessions.

### AI-PBLG: orientation and implementation

Students in the AI-PBLG were organized into subgroups of 4–5 members. A structured orientation session was conducted to introduce them to the functionalities of ChatGPT-4.0, including how to effectively formulate prompts, critically evaluate AI-generated medical information, and use AI outputs for clinical decision-making. A sample case was used to demonstrate live interaction with ChatGPT, after which students completed a brief competency check to ensure baseline understanding.

Each subgroup was then tasked with developing a simulated patient scenario related to CLBP using ChatGPT, followed by generating an evidence-informed evaluation and treatment plan. The AI interaction was designed to encourage active engagement, critical thinking, and learner autonomy. Over the course of one week, each group used ChatGPT to analyze the case, refine clinical hypotheses, and construct a management plan, which was submitted as a group report. These reports were reviewed by the instructor for academic accuracy and used to initiate classroom discussions. Students were then asked to demonstrate key components of their plan on a simulated patient under supervision.

The initial prompt provided to guide students’ use of ChatGPT was:

“I am a physiotherapist treating a patient with CLBP. Please ask me step-by-step questions to help me evaluate and manage this case.”

Additionally, subgroups were encouraged to explore the case further using prompts such as:

- “Which physical assessment tests are most relevant in a patient with CLBP?”
- “How does the biopsychosocial model influence exercise prescription in CLBP?”
- “Which exercises are recommended for patients with CLBP?”
- “How should physiotherapy interventions be modified for a patient with a high restricted motion?”

### AI-PBLG: Instructional structure

- *Classroom Discussions*: Each group summarized their findings, shared AI-generated outputs, and proposed clinical solutions during in-class presentations. The instructor provided immediate, personalized feedback to ensure clinical relevance and accuracy.
- *Clinical Skills Practice*: Following discussions, students practiced patient interviews and physical assessments in simulated bedside scenarios.

The integration of AI with faculty-guided learning aimed to enhance educational outcomes by leveraging both AI scalability and expert supervision. This model promoted active knowledge construction rather than passive information consumption.

### PBLG: orientation and implementation

Students in the PBLG were also divided into subgroups of 4–5 and were provided in advance with a written clinical case on CLBP, relevant textbook chapters, and supplementary reading materials. It was anticipated that they would review the materials independently prior to the subsequent week’s meeting. No AI tools or digital decision support systems were used; all information access remained text-based and instructor-directed.

During the session, the same CLBP case used in the AI-PBLG was introduced via instructor-led presentation. Class discussions were guided by the instructor, who facilitated critical analysis and reasoning based on student responses. The physical examination and therapeutic exercise techniques were demonstrated by the instructor, and selected students participated actively as either demonstrators or observers during hands-on components.

Both AI-PBLG and PBLG received identical course content, instructional materials, and session durations. The only distinction between the groups was the instructional method.

### Outcomes

All participants completed a demographic questionnaire along with a baseline theoretical competency test prior to the start of the intervention. This test is prepared by the instructor who gave the initial educational CLBP course according to the description, reasons, assessment and treatment. The primary endpoints of the study were theoretical knowledge and clinical competence, assessed through the Theoretical Knowledge Test and the Mini-CEX, respectively [16]. Secondary outcomes included the Internet Addiction Test (IAT), the Adult Reading Motivation Scale (ARMS), and the Artificial Intelligence Self-Efficacy Scale (AI-SES) [17–20]. Outcome measures were collected at multiple time points. The theoretical knowledge exam was given at three predetermined time points: (T0) before the initial theoretical session (pre-test), (T1) immediately following the initial theoretical session (immediate post-test), and (T2) after completion of the 2-week intervention (post-test). The IAT, the ARMS, and the AI-SES were administered at two time points: (T1) after the initial theoretical session (immediate post-test) and (T2) after completion of the 2-week intervention (post-test). The Mini-CEX was conducted only at the post-test (T2), following the completion of the intervention.,

### Mini clinical evaluation exercise

The evaluation covered students’ abilities in history taking, physical examination, and clinical reasoning using the validated Mini Clinical Evaluation Exercise **(**Mini-CEX) tool. The instrument features a 9-point rating scale covering seven performance areas: (1) medical interviewing skills, (2) physical examination skills, (3) humanistic qualities/professionalism, (4) clinical judgment, (5) counseling skills, (6) organizational efficiency, and (7) overall clinical competence. Scores range from unsatisfactory (1–3), to satisfactory (4–6), and superior (7–9). All assessments were conducted by the same independent evaluator to ensure inter-rater reliability [16].

### Internet addiction test

To assess baseline technology use and potential overexposure to digital tools, the Internet Addiction Test (IAT) was administered. Each item of the 20-item IAT is scored on a six-point Likert scale ranging from 0 (not applicable) to 5 (always applicable). Problematic internet use is typically identified with scores of 50 or greater, whereas pathological use is indicated by scores of 80 or higher [17, 18].

### Adult reading motivation scale

Intrinsic motivation for academic reading among participants was measured with the 21-item Adult Reading Motivation Scale (ARMS) created by Schutte and Malouff (2007). Items cover multiple domains including self-efficacy, recognition, and reading for broader achievement. The scale uses a 5-point Likert response format, where 1 indicates strong disagreement and 5 indicates strong agreement, producing total scores from 21 to 105. Higher scores indicate greater reading motivation [19].

### Artificial intelligence self-efficacy scale

To evaluate students’ perceived competence in using AI-based technologies, the Artificial Intelligence Self-Efficacy Scale (AI-SES) was employed. Originally developed by Wang and Chuang, this 22-item instrument includes four subscales: assistance, anthropomorphic interaction, comfort, and technological proficiency. It uses a 7-point Likert scale format (1 = strongly disagree, 7 = strongly agree) [20].

### Theoretical knowledge assessment test

The self developed assessment test consisted of 20 multiple-choice questions according to the initial course content given by the expert academician, each offering five answer options. Correct responses were awarded five points, yielding a maximum possible score of 100. A score below 50 was classified as unsuccessful. Students who scored above 50 and met all inclusion criteria were considered eligible for participation. To ensure content validity, the test was reviewed by experts in musculoskeletal physiotherapy.

### Statistical analysis

IBM SPSS Statistics version 24.0 was utilized to perform all statistical analyses (IBM Corp., Armonk, NY, USA). Baseline characteristics, demographic data, and questionnaire responses were analyzed using descriptive statistics. Descriptive statistics included means and standard deviations for continuous variables, and frequencies with percentages for categorical variables. Linear mixed-effects models were applied to each outcome variable, incorporating robust standard errors (Huber-White sandwich estimators). Fixed effects included GROUP (2 levels: AI-PBLG vs. PBLG), TIME (pre-test, immediate post-test, post-test), and the GROUP × TIME interaction. Age and sex were included as covariates. Estimated marginal means (EMMs) with 95% confidence intervals (95% CI) were calculated. Effect sizes for pairwise comparisons were reported as Cohen’s d. P-values underwent adjustment for multiple testing using the Bonferroni correction (pₐ𝖽e). Statistical significance was set at *p* < 0.05. Missing data were handled using a mixed-effects modeling approach, which provides valid estimates under the assumption that data are missing at random (MAR). No imputation techniques were applied, and all available data points were included in the model using maximum likelihood estimation.

## Results

### Participant Characteristics

**Fig. 1:**
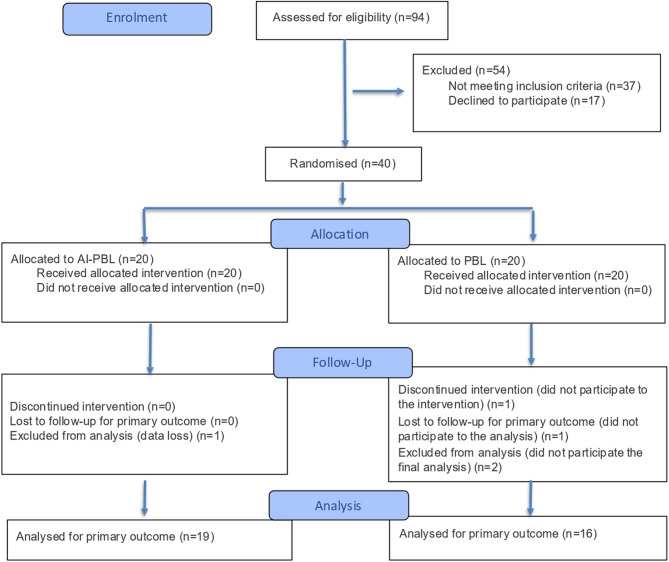
CONSORT 2025 flow diagram of participant enrollment, allocation, follow-up, and analysis

Based on the CONSORT 2025 flow diagram, a total of 94 students were assessed for eligibility. A total of 54 participants were excluded, with 37 failing to meet inclusion criteria and 17 declining participation. Forty students were randomized equally into two groups: AI-PBL (*n* = 20) and traditional PBL (*n* = 20). In the AI-PBL group, one participant was excluded from analysis due to data loss, resulting in 19 participants included in the final analysis. In the PBL group, two participants did not complete the final analysis and were excluded, leaving 16 participants analyzed. (Fig. 1)

The two study cohorts were comparable at baseline across all demographic and clinical variables (*p* > 0.20). For instance, the average age, sex distribution, IAT and ARMS results were similar between PBLG (*n* = 20) and AI-PBLG (*n* = 20) *(p* > 0.05). TKS and AI-SES baseline scores were different between groups (*p* = 0.001, both) (Table 2).

**Table 2:** Baseline demographics and clinical tests of the study groups

Parameter		*N*	PBLG X̄±SD	*N*	AI-PBLG X̄±SD	*p*
Age (years)		20	22.40 ± 2.74	20	22.85 ± 1.53	0.525^U^
TKS		20	45.50 ± 8.09	20	57.10 ± 11.64	0.001^U^
IAT		20	49.00 ± 16.35	20	41.15 ± 14.61	0.118 ^U^
ARMS		20	62.70 ± 14.89	20	61.50 ± 9.54	0.763 ^U^
AI-SES		20	107.05 ± 20.66	20	72.74 ± 22.69	0.001 ^U^
Sex		*N*	%	*N*	%	*p*
	Female	12	60	10	50	0.751^FX2^
	Male	8	40	10	50	

* *p* < 0.05, ^U^: Mann Whitney U test, ^FX2^: Fisher’s Chi Square test, X̄: Mean, *SD* Standard deviation, *AI-PBLG* Artificial intelligence–supported problem-based learning group, *PBLG* Traditional problem-based learning group, *IAT* Internet Addiction Test, *ARMS* Adult Reading Motivation Scale, *AI-SES* Artificial Intelligence Self-Efficacy Scale, *TKS* Theoretical knowledge scores

### Within and between-group comparison of change scores and overall model effects

Following the two-week instructional intervention, significant differences emerged between the AI-PBLG (*N* = 19) and the PBLG (*N* = 16) across several secondary outcomes.Each outcome was assessed at two time points (baseline and post-intervention), and Δ mean scores represent the magnitude of change from pre- to post-intervention within each group (Table 3).

**Table 3:** Within- and between-group changes in theoretical knowledge scores across time points in the AI-PBL and traditional PBL groups

Time	AI-PBLG X̄	PBLG X̄	Pooled SD	Cohen d	**p
Pre-test (T0)	57.6	46.8	10.2	1.07	0.003
Immediate post-test (T1)	64.2	60.6	9.7	0.37	0.268
Post-test (T2)	81.8	69.4	11.5	1.08	0.003
*P_T1−2_ (Cohen d)	0.031 (0.54)	*p* < 0.001 (1.73)			
*P_T1−3_ (Cohen d)	*p* < 0.001 (1.7)	*p* < 0.001 (1.76)			
*P_T2−3_ (Cohen d)	*p* < 0.001 (3.14)	0.006 (0.77)			

X̄:Mean, *SD* Standard Deviation, *AI-PBLG* Artificial intelligence–supported problem-based learning group, *PBLG* Traditional problem-based learning group, *P*_*T0−1*_Pre-Immediate post test, *P*_*T*__*0-2*_ Pre-Post test, *P*_*T*__*1-3*_ Immediate post-post test

*p: Within groups analyses (group×time interaction), **p: Between groups analyses, *p* < 0.05

For IAT scores, both groups showed a slight increase from pre- to post-test. AI-PBLG exhibited a modest improvement (ΔX̄=4.7, *p* = 0.323, *d* = 0.23), while PBL showed a slightly larger, yet still non-significant improvement (ΔX̄=6.5, *p* = 0.092, *d* = 0.43). For IAT, no significant group differences were detected between the AI-PBLG and PBLG participants. The mean change in the AI-PBLG was ΔX̄=4.7 (SD = 20.3), while in the PBLG it was ΔX̄=6.5 (SD = 14.9), yielding a negligible between-group effect (Cohen’s *d*=–0.10, *p* = 0.771).

In contrast, the ARMS scores significantly improved in both groups, with a more pronounced effect in the AI-PBLG with a large effect size (*d* = 1.31, *p* < 0.001). Meanwhile, the PBLG group improved a moderate effect size (*d* = 0.64, *p* = 0.017). For ARMS, although both groups showed improvements over time (AI-PBLG ΔX̄=12.7, SD = 9.7; PBLG ΔX̄=9.8, SD = 15.2), the difference between groups did not reach statistical significance (Cohen’s *d* = 0.24, *p* = 0.495).

The most substantial difference was observed in the AI-SES. The AI-PBLG exhibited a significant difference, representing a very large effect size (*d* = 1.70, *p* < 0.000001). Conversely, the PBLG group experienced a non-significance (*d*=–0.26, *p* = 0.327). The AI-PBLG exhibited a significant increase in self-efficacy scores (ΔX̄=19.8, SD = 11.7), whereas the PBLG group experienced a decrease (ΔX̄=–3.9, SD = 15.0). This contrast resulted in a very large between-group effect size (Cohen’s *d* = 1.78) and reached a high level of statistical significance (Welch’s t = 5.00 and *p* = 0.00003).

Mini-CEX scores, following the two-week intervention, were higher in the AI-supported PBL group compared to the traditional PBL group. Although the AI-PBL group outperformed the control group numerically, the between-group difference was not statistically significant (*p* = 0.236), with an effect size ranging from small to moderate (Cohen’s *d* = 0.42).

The linear mixed-effects models showed significant main effects for GROUP [F(X, Y) = 23.45, *p* < 0.001, ηp²=0.08] and TIME [F(X, Y) = 63.12, *p* < 0.001, ηp²=0.43] indicating overall improvement across the study period and consistent differences between groups. The GROUP × TIME interaction was not significant for most outcomes [F(X, Y) = 2.5, *p* = 0.088, ηp²=0.02], suggesting similar temporal patterns of change. Targeted comparisons confirmed no significant differences between groups in Internet Addiction or Reading Motivation (*p* > 0.05), although AI-PBLG showed slightly greater improvements. In contrast, AI Self-Efficacy was significantly higher in the AI-PBLG (*p* = 0.003, *d ≈* 1.78), indicating greater confidence and competence in AI use among students directly interacting with AI tools.

### Theoretical knowledge outcomes

Between-group comparisons showed that the AI-PBL group (*N* = 19) consistently outperformed the traditional PBL group (*N* = 16) at each time point (P_T1−2_, P_T1−3_,P_T2−3_). The between-group difference was small at post-test (Cohen’s *d* ≈ 0.54, *p* = 0.031) but became large at 2-week follow-up (Cohen’s *d* > 1.70).

In AI-PBLG, theoretical scores there was a significant increase in scores from baseline to post-treatment (*ΔM* = 6.6, *p* = 0.031, *d* = 0.54), and from pre to 2-week follow-up (*ΔM* = 24.2, *p* < 0.001, *d* = 1.70). A very large effect was also observed between post and 2-week follow-up (*ΔM* = 17.6, *d* = 3.14). PBLG also showed significant gains between pre and post (*ΔM* = 13.8, *p* < 0.001, *d* = 1.73) (Table 4).

**Table 4:** Between-group comparisons of theoretical knowledge scores across three time points Within-group comparisons of theoretical knowledge scores across three time points

**Outcome**	**Group**	**Pre-test** **(X̄ ±SD)**	**Post-test** **(X̄ ±SD)**	***p***	**d**	**Δ ** **X̄ ±SD**	*d	*p
IAT	AI-PBL	40.8±17.1	45.5±14.1	0.323	0.23	4.7±20.3	−0.1	0.771
	PBL	48.4±14.9	54.8±18.4	0.092	0.43	6.5±14.9		
ARMS	AI-PBL	61.4±9.8	74.2±3.9	p<0.001	1.31	12.7±9.7	0.24	0.495
	PBL	63.5±15.5	73.2±6.8	0.017	0.64	9.8±15.2		
AI-SES	AI-PBL	70.7±21.4	91.6±21.7	p<0.001	1.7	19.8±11.7	1.78	0.00003
	PBL	109.6±19.0	104.6±18.2	0.327	−0.26	−3.9±15.0		
Mini-CEX	AI-PBL	NA	38.0±13.1	0.236	0.42	NA	NA	NA
	PBL	NA	31.3±18.5			NA	NA	NA

*p* < 0.05; *p: Between groups test, *IAT* Internet Addiction Test, *ARMS,* Adult Reading Motivation Scale, *AI-SES* Artificial Intelligence Self-Efficacy Scale, *Mini-CEX* Mini Clinical Evaluation Exercise, *AI-PBLG* Artificial intelligence–supported problem-based learning group, *PBLG* Traditional problem-based learning group, d: Cohen’s d, *d: Between groups test, X̄:Mean, *SD* Standard Deviation

## Discussion

The purpose of this randomized controlled study was to evaluate and compare the effects of an AI-supported PBL module and a traditional PBL approach on theoretical knowledge, AI self-efficacy, reading motivation, and internet addiction among undergraduate physiotherapy students. The AI-PBL group outperformed the traditional PBL group in several domains, particularly in theoretical knowledge retention and AI self-efficacy. Notably, very large effect sizes were observed in knowledge gains from pre- to post-test (Cohen’s *d* = 1.70) and from post-test to 2-week follow-up (Cohen’s *d* = 3.14), indicating enhanced understanding and consolidation. However, mini-CEX did not reveal any significant difference over time in terms of reading motivation and internet addiction. These results support the integration of generative AI tools like ChatGPT into clinical education, especially for complex topics such as CLBP. While both groups showed immediate gains, only the AI-PBL group demonstrated continued improvement at follow-up in terms of perceived competence in applying AI and engaging with academic reading, suggesting that sustained learning was promoted by active, iterative interaction with the AI tool.

AI-supported learning environments have been reported to offer numerous cognitive and clinical benefits, such as enhanced learner engagement, critical thinking, increased participation in the learning process, tailored virtual simulations, and case-based feedback mechanisms [8, 9, 21, 22]. Gupta et al. highlighted that generative AI could help to address challenges such as information overload, limited clinical exposure, and learning gaps. Additionally, AI-supported education has been shown to reduce study time by approximately 27%, thus enhancing learning efficiency [23, 24]. Hui et al. found that students who received ChatGPT-assisted instruction in medical education demonstrated higher post-education performance and deeper conceptual understanding [8]. While such advantages have been widely discussed in medical and nursing education, experimental evidence in the context of physiotherapy remains limited. The present study aims to contribute to the literature by implementing an AI-supported PBL approach tailored to the topic of CLBP in physiotherapy education.

Jackson et al. reported that more than 90% of medical students had not received formal AI education, yet approximately 75% expressed a desire for structured training, particularly in clinical applications and ethical issues. Similarly, recent research in nursing education has shown that AI literacy and an innovative mindset play a crucial role in shaping students’ career development and perceived self-efficacy [25]. In a large-scale study conducted by El-Sayed et al. involving 596 undergraduate nursing students, AI literacy and innovative thinking were found to significantly predict students’ confidence in managing their careers. The study also emphasized that integrating AI competencies with forward-thinking pedagogical strategies could create strong synergy in preparing students for rapidly evolving healthcare environments [26]. However, despite the growing role of AI in education, it remains underexplored in the physiotherapy domain. Considering that physiotherapists are expected to work in increasingly complex and technology-intensive clinical settings, there is a critical need to examine how AI literacy and innovative thinking affect physiotherapy students’ academic engagement, clinical reasoning, and professional identity formation. Accordingly, the main objective of our study is to demonstrate that integrating AI-based educational models into physiotherapy education can enhance theoretical knowledge, reflective thinking, and self-efficacy development. Although physiotherapy students’ attitudes toward AI education are not yet clearly defined, the gap between interest and available educational opportunities points to a significant shortcoming in current curricula. The AI-PBL intervention developed in this study seeks to address this gap by offering an innovative and structured educational solution.

In a large-scale meta-analysis including 51 studies, Wang et al. reported that the use of ChatGPT led to a significant improvement in students’ learning performance (Hedge’s *g* = 0.867). The study further emphasized that these gains became more pronounced when ChatGPT was integrated into structured instructional strategies such as PBL, and were associated with higher-order cognitive outcomes (*g* ≈ 0.46) [9]. Particularly in disciplines like physiotherapy education, which demand practical application and clinical reasoning, the student-centered nature of PBL and its reliance on experiential learning are known to support deep learning. Korpi et al. argued that PBL enhances both individual reflective skills such as information seeking and creative learning, and collaborative reflection through peer interaction and instructor guidance. In this sense, PBL presents a more effective pedagogical model compared to traditional experiential learning approaches [27].

In our study, theoretical knowledge scores significantly improved in both groups following the initial instructional session. This multiple-choice examination was systematically developed to evaluate factual knowledge, conceptual comprehension, and clinical reasoning in relation to CLBP, encompassing its clinical presentation, diagnostic findings, physical examination, and management strategies. Therefore in this test, higher scores correspond to higher educational knowledge of students in CLBP. However, the AI-PBL group demonstrated not only a meaningful gain from pre-test (T0) to immediate post-test (T1) (Cohen’s *d* = 0.54), but also an exceptionally large improvement from immediate post-test (T1) to the 2-week retention/post test (T2) (Cohen’s *d* = 3.14). This very large effect size suggests that students in the AI-supported group engaged in sustained learning beyond the structured instructional period. These learning gains may have stemmed from the interactive experience with ChatGPT, which encouraged questioning, scenario generation, and self-directed learning. In contrast, while the traditional PBL group exhibited a strong improvement from pre-test to immediate test (Cohen’s *d* = 1.73), their knowledge consolidation from post-test to retention test was lower (Cohen’s *d* = 0.77), indicating potentially slower reinforcement of knowledge compared to the AI group.

Additionally, our study found that ChatGPT-supported PBL sessions contributed not only to the development of clinical reasoning but also to increased self-efficacy in using generative AI tools effectively and ethically. Shorey et al. highlighted ChatGPT’s potential to enhance learning outcomes through real-time feedback, personalized instruction, and improved student engagement—especially for learners with limited communication skills or clinical confidence. The authors also emphasized that ChatGPT promotes deeper learning by supporting self-directed exploration and scenario-based reasoning, which are core features of AI-enhanced PBL [22]. While our study observed significantly greater gains in theoretical knowledge in the AI-PBL group, there was no statistically significant difference between the groups in clinical competence as measured by the Mini-CEX, although the AI-PBL group had numerically higher scores. One possible explanation for the absence of a group difference in Mini-CEX outcomes is the shared practical component delivered to both groups during the first week of the intervention. Since both groups received the same practical training before participating in their respective PBL sessions, this may have equalized baseline clinical competencies and provided similar opportunities for skill acquisition.

Prior research in nursing and medical education has shown that PBL curricula enhance intrinsic reading motivation by linking academic reading directly to clinical problem solving and encouraging learner ownership [28, 29]. Similarly, studies using chatbot-based microlearning environments report that AI-supported learners demonstrate higher intrinsic motivation and engagement, particularly when the system fosters active reflection and learner input [30, 31]. Our study’s outcomes are congruent with the existing literature, as the AI-PBL group showed greater gains in reading motivation, suggesting that active interaction with ChatGPT may have enhanced students’ perceived autonomy and interest in engaging with academic materials. Although both groups demonstrated improvements in ARMS scores, the motivational impact of AI-supported PBL was greater, though the difference did not reach statistical significance. Importantly, concerns regarding passive learning or reduced critical reading with unguided AI use [32] were addressed in our study through a structured, group-based design that encouraged students to actively question, reflect on, and validate AI-generated content. This approach may have strengthened their independent motivation to read and verify information. Additionally, although ChatGPT inherently requires internet access, our results did not indicate any increase in internet addiction, suggesting that its educational use may not pose additional behavioral risks.

Hui et al. evaluated a ChatGPT-assisted PBL method and found that improvements in theoretical knowledge and clinical skills could be achieved within just three days of training [8]. In contrast, our study extended the assessment to a two-week follow-up period, offering insights into the sustainability of learning gains. Furthermore, our study provided a more comprehensive perspective by incorporating multidimensional outcome variables such as internet addiction, AI self-efficacy, and reading motivation allowing for a deeper understanding of how generative AI affects learning behaviors. Previous studies by Hui and Hao have also identified certain limitations of ChatGPT-supported learning, such as occasional misinterpretation of medical terminology and the tendency to provide overly general responses in ambiguous clinical scenarios. These issues highlight that generative AI cannot replace human judgment and underscore the importance of educator supervision during AI-assisted learning [8, 33]. Our study addressed these concerns by embedding AI interactions within supervised, small-group discussions, which encouraged collaborative evaluation of ChatGPT-generated content and helped mitigate ethical uncertainties. While the findings of both studies support the use of ChatGPT in clinical education, our results suggest that its effectiveness and safety can be further enhanced when integrated into a reflective and collaborative learning model supported by peer dialogue and guided self-assessment.

In accordance with all these findings from our study, we recommend to the academicians, to use these technologies as supportive methods in education. AI technologies can not be the only source for sure for the education in the field of physiotherapy and rehabilitation, but using the advantages of these systems in case analysis, clinical patient analysis and to review the treatment options can definitely be time saving, effective and comprehensive. While teaching about the diseases, assessment and treatment methods in physical therapy are the duties of the physiotherapy teachers, the education can be reinforced by sample cases created through these systems, treating the AI system as a patient, asking questions and getting answers from it, and therefore creating meaningful, real time clinical scenarios. Rather than fearing that students may misuse these systems to simplify learning, avoid in-depth study, or disrupt traditional instructional methods, educators should focus on teaching students how to effectively engage with such tools, guiding them to formulate appropriate clinical questions and critically evaluate responses to identify accurate treatment solutions. Given that technological systems may also lead to negative outcomes such as technology addiction, avoidance of studying, and academic dishonesty, it is the responsibility of academic professionals and educators to teach appropriate usage practices and clearly define ethical guidelines instead of avoiding inevitable, worldwide common technologies.

There are a number of limitations in this study that should be acknowledged. Notably, the relatively small sample size may have constrained the statistical power to identify between-group differences, especially regarding secondary outcomes. The use of a single post-test administration for clinical competence (Mini-CEX) also constrains the interpretation of long-term or progressive skill acquisition. Another limitation is the lack of objective tracking of student engagement with learning materials. In the AI-PBL group, the amount of time spent interacting with ChatGPT was not recorded, nor was the time spent using academic resources in the traditional PBL group. Future studies should include behavioral measures, monitor time spent on learning resources, and apply extended follow-up to confirm and build upon these results.

## Conclusion

This educational approach in physiotherapy, which blends AI-human collaboration within a structured PBL framework, highlights the potential of generative AI to become a meaningful pedagogical aid when students are guided to critically engage with its content through academic supervision. When aligned with safe practice principles and paired with strategies that support learning motivation, AI may contribute positively to the development of knowledge, skills, and clinical reasoning in physical therapy and rehabilitation education. By knowing the fact that these systems cannot substitute for the expertise and guidance of professional academic instructors; but they can serve as valuable educational tools by integrating clinical scenarios, promoting clinical reasoning, and facilitating interactive case-based learning within the classroom environment especially in the practical, laboratory courses. In this light, AI-supported PBL may be seen not only as a means to enhance cognitive outcomes but also as a promising avenue to cultivate professional dialogue grounded in trust, empathy, and responsibility key values for healthcare professionals in the digital age.

## Supplementary Information

Supplementary Material 1.

Supplementary Material 2.

## Data Availability

Dataset available on request from the authors.

## Abbreviations

AI: Artificial Intelligence
AI-PBL: Artificial intelligence–assisted problem-based learning
AI-PBLG: Artificial intelligence–supported problem-based learning group
AI-SES: Artificial intelligence self-efficacy scale
ARMS: Adult reading motivation scale
CLBP: Chronic low back pain
IAT: Internet addiction test
Mini-CEX: Mini clinical evaluation exercise
PBL: Problem-based learning
PBLG: Traditional problem-based learning group
