# Supplementary material for: Effects of artificial intelligence based physiotherapy educational approach in developing clinical reasoning skills: a randomized controlled trial
Source: BMC Med Educ. 2025 Oct 9;25:1378. doi: 10.1186/s12909-025-07926-w (PMC12512892; doi:10.1186/s12909-025-07926-w)
Supplement: Supplementary file 1 — Supplementary Material 1. [file 12909_2025_7926_MOESM1_ESM.pdf]

Lumbar Disc Herniation Case Report

A 40-year-old right-dominant housewife, height 162 cm, weight 78 kg, BMI: 29.7 kg/m² (overweight). The patient presented with complaints of pain starting in the lower back and radiating to the hip and leg, worsening with prolonged standing, or sitting, and disturbing her sleep at night. She had intermittent low back pain for two years and experienced episodes of locking in her back twice. About a month and a half ago, she felt a sudden strain in her back while lifting her child. Her pain worsened, began to radiate to her leg, and was accompanied by restricted spinal movement. She consulted an orthopedic and traumatology specialist. Physical examination and MRI revealed an extruded disc at L4-L5 and a protruded disc at L5-S1. She was referred to orthopedic rehabilitation.

Assessment

Medical History: Two children delivered via cesarean section (8 and 3 years ago). Previously treated conservatively with medication for disc herniation.

Family History: Hypertension in both parents.

Habits: Goes walking in a nearby park twice a week.

Medications: Occasionally uses painkillers and NSAIDs for pain relief.

Inspection and Palpation: Right lateral flexion of the trunk was observed. Muscle tightness and tenderness were noted in the lumbar region and paravertebral muscles, as well as in the hip area (piriformis, iliopsoas, tensor fasciae latae, hamstrings).

Pain Assessment: The patient reported pain radiating from the lower back to the left hip and leg, increasing during forward bending and rotational movements. Pain intensity was rated using the Visual Analog Scale (VAS) as 4 at rest, 8.5 during activity, and 5.4 at night.

Posture Analysis: Anterior, posterior, and lateral posture assessments revealed lumbar flattening, posterior pelvic tilt, shoulder protraction, forward head posture, second-degree pes planus, and right lateral trunk flexion.

Range of Motion (ROM) Assessment of Lumbar Spine: Lumbar spine flexion, extension, lateral flexion, and rotation were observed. Movements were limited due to pain and assessed observationally within the pain-free range.

Trunk and Lower Extremity Muscle Strength Assessment: Due to pain and radicular symptoms, no strength test was conducted in the first session. After two weeks, trunk muscles were evaluated. Hip extension could not be tested due to pain. Left knee flexion and hip external rotation were scored 4/5; other muscles were 4/5. Trunk extensors, lateral trunk flexors, obliques, abdominal muscles, and lower limb muscles were tested manually, with trunk flexors/extensors scoring 3+, hip/knee 4, and distal muscles 5.

Muscle Shortness Tests: Flexibility tests indicated tightness in the hip flexors, hamstrings, and tensor fasciae latae.

Special Tests for Lumbar Region: Straight Leg Raise (SLR), Lasegue, and Bragard tests were positive, consistent with L4-L5 and L5-S1 disc herniation seen on MRI.

Reflex Assessment: Achilles reflex was tested for S1 root compression; response was normal.

Gait Analysis: Observational analysis revealed a compensatory gait pattern with trunk lateral flexion, short step length, and decreased arm swing.

Functional Assessment: Oswestry Disability Index (ODI) scored 62%, indicating severe functional limitation due to back pain.

Quality of Life: Assessed using the Short Form-36 (SF-36); several subdomains showed impaired quality of life.
